# Challenges in diabetes management in Indonesia: a literature review

**DOI:** 10.1186/1744-8603-9-63

**Published:** 2013-12-03

**Authors:** Pradana Soewondo, Alessandra Ferrario, Dicky Levenus Tahapary

**Affiliations:** 1 Division of Metabolism and Endocrinology, Department of Internal Medicine, Faculty of Medicine University of Indonesia, Dr. Cipto Mangunkusumo Hospital, Jakarta, Indonesia; 2 LSE Health, London School of Economics and Political Science, Houghton Street, WC2A 2AE, London, UK

**Keywords:** Diabetes mellitus, Diabetes costs, Diabetes complications, Indonesia

## Abstract

**Background and objectives:**

The expanding diabetes epidemic worldwide could have potentially devastating effects on the development of healthcare systems and economies in emerging countries, both in terms of direct health care costs and loss of working time and disability. This study aims to review evidence on the burden, expenditure, complications, treatment, and outcomes of diabetes in Indonesia and its implications on the current health system developments.

**Methods:**

We conducted a comprehensive literature review together with a review of unpublished data from the Ministry of Health and a public health insurer (*Askes*). Studies presenting evidence on prevalence, incidence, mortality, costs, complications and cost of complications, treatment, and outcomes were included in the analysis.

**Results:**

A limited number of international, national and local studies on the burden and cost of diabetes in Indonesia were identified. National survey data suggests that in 2007 the prevalence of diabetes was 5.7%, of which more than 70% of cases were undiagnosed. This estimate hides large intracountry variation. There was very limited data available on direct costs and no data on indirect costs. The most commonly-identified complication was diabetic neuropathy.

**Discussion:**

There were a number of limitations in the data retrieved including the paucity of data representative at the national level, lack of a clear reference date, lack of data from primary care, and lack of data from certain regions of the country.

**Conclusions:**

If left unaddressed, the growing prevalence of diabetes in the country will pose a tremendous challenge to the Indonesian healthcare system, particularly in view of the Government’s 2010 mandate to achieve universal health coverage by 2014. Essential steps to address this issue would include: placing diabetes and non-communicable diseases high on the Government agenda and creating a national plan; identifying disparities and priority areas for Indonesia; developing a framework for coordinated actions between all relevant stakeholders.

## Background

With a population of 237.6 million people in 2010 [1], Indonesia is the world’s fourth most populated country. It also has the seventh largest number of diabetic patients (7.6 million), despite relatively low prevalence (4.8% including both diabetes type 1 and 2 in individuals aged 20–79 years) in 2012 [2].

The country is in the middle of a demographic and epidemiological transition. In 2009, life expectancy at birth was 68 years, which is slightly higher than the Southeast Asian regional average of 65 years [3]. The fertility rate decreased from 3.1 in 1990 to 2.5 in 2000, reaching 2.1 in 2009 [3]. Both adult mortality and under-five mortality are below regional averages (190 deaths in Indonesia vs. 209 deaths in the region among adults 15–59 years old per 1000; 39 vs. 59 deaths among children under five per 1000 live births) [3]. The Indonesian epidemiological transition is proceeding rapidly in comparison to the regional average. In 2008, 41% (49% regional average) of the total years of life lost (YLL) was due to communicable diseases while 45% (36% regional average) were due to non-communicable diseases [3].

However, marked geographical variations exist. While infectious diseases and child mortality are still very prevalent in the eastern provinces of Indonesia, Java and Bali are starting to experience a higher burden of non-communicable diseases (NCDs) [4].

The rising prevalence of diabetes has become a major problem worldwide and affects more than 132.2 million in the Western Pacific region (more people than in any other region) [2]. Non-communicable diseases are estimated to account for 63% of all deaths in Indonesia [5]. Cardiovascular disease contributed to 30% of the total number of deaths followed by cancers (13%), and diabetes (3%) [5]. The epidemiological and nutritional transitions have played a major role in these trends [6].

Indonesia’s struggle to develop a responsive healthcare system is exacerbated by an environment where health insurance coverage is incomplete. The Government aims to achieve universal health coverage by 2014 by progressively covering the remaining 139.9 million uninsured citizens through *Askeskin/Jamkesmas*[7].

The main government health insurance programmes are *Askeskin/Jamkesmas,* which is the national health insurance scheme for the poor or nearly poor (76.4 million beneficiaries representing 32% of the population in 2010). *Askes* provides health insurance for civil servants and retired army forces (16.5 million beneficiaries representing 7% of the population in 2010). *Jamsostek* provides coverage for formal sector workers (5.0 million beneficiaries representing 2% of the population in 2010) [8]. Meanwhile, about 3% of the population is covered by private health insurance [4].

Due to the potentially devastating effects of the diabetes epidemic to the development of the Indonesian health system and economy (both in terms of direct health care costs and loss of working time and disability), this study aims to review evidence on the burden, expenditure, complications, treatment, and outcomes of diabetes in Indonesia and its implications on the current health system developments.

To our knowledge, no such study had been published at the time of writing. Reviewing available evidence at regular intervals is crucial to assess the situation and to inform policies and programme implementation. This is particularly relevant at this point in time, as the Government of Indonesia is preparing its national plan for diabetes. This literature review thus aims to address this important literature gap by reviewing and critically assessing available evidence, and making recommendations on areas of diabetes management which need to be strengthened.

## Methods

We reviewed available evidence and summarised available data sources on: the prevalence of type 1 and type 2 diabetes and gestational diabetes; the incidence, direct and indirect costs of diabetes; prevalence of complications (diabetes retinopathy, neuropathy, nephropathy, chronic kidney disease, and vascular complications including diabetic foot); cost of complications; treatment regimens and use of renal transplantation and impact outcomes such as HbA1c levels; frequency of checks for complications and glucose monitoring. Further, we reviewed available national guidelines for diabetes, available policies and national programmes, and searched for any available evidence on the impact of smoking, tuberculosis (TB), HIV, and fasting during Ramadan on health outcomes for diabetic patients. Where such information was available, we distinguished between diabetes type 1 and 2.

A comprehensive literature review on diabetes care management was conducted in February 2012. The following key words were used ((diabetes[Title/Abstract] OR "chronic kidney" OR "renal disease") AND Indonesia[Title/Abstract]) OR (("Diabetes Mellitus"[Mesh] OR "Diabetes Mellitus, Type 2"[Mesh] OR "Diabetes Mellitus, Type 1"[Mesh]) AND "Indonesia"[Mesh]) in PubMed. Diabetes, prevalence, treatment, complications, chronic kidney, renal disease, direct cost, indirect cost, health insurance, health system, Indonesia were used in Google and local journals. We did not put any time limits or language restrictions on our search and all articles identified up to February 2012 were included.

Additional articles were identified from the references in the retrieved literature.

We also retrieved unpublished data from the Ministry of Health Republic of Indonesia and *Askes*. In addition, we also included presentations from symposia on diabetes.

Studies presenting evidence (whether primary or secondary) on prevalence, incidence, mortality, costs, complications and cost of complications, treatment, outcomes (e.g. blood glucose control) were included in the analysis.

If data on costs were only reported in Indonesian Rupee (IDR), we converted the amounts into USD$ using the corresponding historical conversion rate from the OANDA website (http://www.oanda.com/currency/historical-rates/).

## Results

We retrieved 93 studies in PubMed: 69 were excluded through title screening, one was excluded through abstract screening because they did not meet inclusion criteria. A total number of 23 peer-reviewed papers were included in the analysis (Table 1). Four additional peer-reviewed papers were identified through Google search. Another 11 articles were identified through reference searching in other articles. This was complemented by grey literature such as reports and presentations.

**Table 1:** Literature

**Area of diabetes management**	**Number of references retrieved**	**References**
Prevalence	8	[9-16]
Incidence	0	-
Mortality	1	[17]
Costs	3	[18-20]
Complications	14	[18,21-32] and unpublished observations from Yunir 2008^i^
Costs of complications	5	[20,22,33-35]
Treatment	6	[21,26,35-38]
Outcomes	7	[18,21,26,36,39-41]

### Data sources

A limited number of data sources on the burden and management of diabetes in Indonesia were identified. These included international studies, national surveys, and studies.

#### ***Diab care***

DiabCare Asia is an international collaboration between Novo Nordisk Asia Pacific, Singapore, BioRad Pacific, Hong Kong and diabetes associations in participating Asian countries. The aim of this partnership is to collect evidence on disease pattern, management, control status, and complications of diabetes in the Asian diabetes population.

DiabCare in Indonesia was conducted in 1997, 1998, 2001, 2003, and 2008 to estimate the prevalence of diabetes and its complications and to investigate treatment outcomes [26]. In addition, DiabCare 2003 and 2008 also evaluated the quality of life of patients with diabetes.

#### ***International diabetes management practices study (IDMPS)***

The IDMPS is one of the largest population-based studies of diabetes patients in developing countries. This cross-sectional study aimed to estimate resource use for diabetes type 2 and risk factors for hospitalisation, inpatient days, emergency visits, and absenteeism in 24 countries in Africa, Asia, Latin America and the Middle East [18].

#### ***National basic health research (Riskesdas)***

The Riskesdas survey was commissioned by the National Institute of Health and Research (NIHRD) to provide evidence for policy-makers on key health areas such as health status, nutrition status, health environment, health attitude, and various health service utilisation aspects. Evidence collected includes nationally representative indicators together with some district/city level indicators based on a sample of 258,284 households comprising 972,989 individuals. In 2007, for the first time blood glucose was measured allowing for the estimation of diabetes prevalence. For this purpose, blood samples were collected from 24,417 urban residents aged 15 and over [16]. The next round of data collection as part of Riskesdas is planned for 2013.

#### ***A1CHIEVE***

A1chieve was a large multicountry observational study on the use of insulin in DMT2 patients in real-life. The aim was to evaluate the safety and effectiveness in routine clinical practice between 2009 and 2010 of three insulin analogues manufactured by Novo Nordisk. The study recruited 66,726 people across 3,166 centres in 28 countries covering the four continents (Asia, Africa, South America, and Europe). The primary aim of the study was to assess the adverse event profile of the three insulins in routine clinical practice, including rates of hypoglycaemia. In addition, effectiveness (HbA1c, fasting plasma glucose, and postprandial plasma glucose) and patient quality of life outcomes were measured [42].

#### ***Askes***

Most direct cost information available is unpublished data from the health insurer *Askes*.

#### ***Jakarta primary non-communicable disease risk factors surveillance (Jakarta NCD-S)***

The Jakarta NCD-S study is the result of a collaboration between the Ministry of Health and the University of Indonesia started in 2006 [43]. Data collected include the prevalence of diabetes and its risk factors and complications [43-45].

#### ***Indonesian renal registry***

A national registry for end stage renal disease has been established by the Indonesian Society of Nephrology (PERNEFRI) to collect information on the prevalence and incidence of end stage renal disease [22,34,46].

#### ***Studies***

A few local cross-sectional studies on the prevalence of diabetes and its complications were identified. These studies investigated the prevalence and risk factors in a remote island in the eastern part of the country [12]; prevalence in population sub-groups [9]; prevalence and risk-factors for diabetes in northern Jakarta [15]; childhood diabetes cases in a hospital [13]; prevalence of decreased renal function among diabetes type 2 patients [25]; prevalence of diabetes foot [24]; and the incidence and prevalence of end-stage renal disease [22,34].

Evidence from secondary sources identified in this study and the authors’ own knowledge suggest that an unknown number of local studies on the prevalence of diabetes in Indonesia remain unpublished. We could identify some of these studies from secondary sources; however, information on the study methodology and other important parameters such as time and location were often missing from secondary sources limiting the use and comparability of such data.

Local studies are complemented by international efforts such as the PAD-SEARCH, an international study on the prevalence of peripheral arterial disease in Asian type 2 diabetes patients [23] and multi-country studies on the prevalence of complications and outcomes of diabetes type 1 in children [21] and type 2 [36] in youth.

Other articles presented findings from secondary data sources: Riskedas 2007 [10,11], diabetes complications and outcomes from DiabaCare 2008 survey [26], and a review reported results from unpublished epidemiological studies [14].

In addition to data from *Askes*, a few local studies addressing direct cost of diabetes were also identified [19,20,33,47].

### Diabetes prevalence

According to the International Diabetes Federation (IDF), the national prevalence of diabetes in Indonesia was 4.8% in 2012 (the international comparative prevalence was 5.1% in 2012) [2]. More than half of all diabetes cases (58.8%) were underdiagnosed in 2012 [2]. The proportion of urban and rural cases was almost the same, although slightly higher in urban areas (1.1 urban : rural ratio in 2012) and it is expected to increase to an estimated 1.6 urban : rural ratio by 2030 [2].

Data from the 2007 Indonesian National Basic Health Research (*Riskesdas*), found a prevalence of diabetes of 5.7%, with more than 70% undiagnosed diabetes cases [16] (Table 2). However, this estimate hides large variation within the country with the lowest prevalence in the province of East Nusa Tenggara (1.8%) and the highest in the provinces of West Kalimantan and North Malaku (11.1%) [16].

**Table 2:** Prevalence of diabetes mellitus in Indonesia

**Prevalence of DM**					
**Estimate**	**Study year**	**Sampling frame and sample**	**Study design**	**Diagnostic test and diagnostic criteria**	**Reference**
19.6%, men 21.9%, women 18.2%, n = 97, N = 495	2008	Sampling frame: Suburban population of Ternate, a small remote island in Eastern Indonesia	Cross-sectional survey	Diagnostic test: FPG	[12]
Peak age group was 50–59 years old, followed by 40–49 years old, and 60–69 years old		Sample: 495 individuals, 187 (37.8%) men and 308 (62.2%) women, 20–84 years old		Criteria: ADA 1997 FBG (>126 mg/dl)	
5.7%, 1.5% diagnosed, 4.2% undiagnosed 6.4% women, 4.9% men	*Riskesdas* 2007	Sampling frame: 33 provinces, urban areas	Cross-sectional survey	Diagnostic test: FPG and OGTT	[11,16]
		Sample: 24,417 urban residents, >15 years old and not pregnant		Criteria: WHO 1999 and ADA 2003 (> = 200 mg/dl)	
1.7% (1982), 5.7% (1993), 12.8% (2001) in Jakarta1.5% (NA), 5.4% (NA) in Ujung Pandang, 6.1% in Mandado (1980s), 1.5% Bali (NA) East Java rural and urban areas, 1.47% (NA)	1980s, 1982, 1983, 1995, 2001	Sampling frame: Various urban and rural areas	NA	Diagnostic test: NA	Data from local unpublished studies, results reported in [14,48] and unpublished observations from Soebardi 2011
		Sample: NA		Criteria: NA	
Rural area of Ende, found a much lower prevalence of 1.56% (NA)					
Asymptomatic cases of diabetes	1997 population screening, 2000 clinical screening	Sampling frame: A group of government employees and retired military officers	Population screening and clinical screening	Diagnostic test: Population screening with OGTT. Clinical screening with FBG and OGTT	[9]
Population screening: 5.42%, n = 51 both sexes 7.42%, n = 21 women 4.6%, n = 30 men					
		Sample: Population screening: 941 (290 women, 651 men), clinical screening: 907 (483 women, 424 men)		Criteria: WHO 1999 criteria	
Clinical screening: 17.08%, n = 155 both sexes, 16.14%, n = 78 women, 18.16%, n = 77 men					
1.63%, n = 44, N = 2,704, 18 (40%) had previously been diagnosed but only 50% of them were on treatment, 1.8%, n = 23 men, 1.5%, n = 21 women	Study year: December 1981-June 1982	Sampling frame: Koja Utara subdistrict, Tanjungpriok, Northern Jakarta	NA	Diagnostic test: OGTT	[15]
				Criteria: WHO 1980 criteria	
		Sample: 2,704, > = 15			
Peak age was 55–64 for both men and women					
0.2% (Semarang), 0.26% (Surabaya)	Study year: 1974, 1977, 1973-1977	Sampling frames: Cities of Semarang (1974), Surabaya (1977), one clinic (1973–1977)	NA	Diagnostic test: NA Criteria: NA	Review of previous study plus data from the authors’ clinic [13]
28 patients with insulin-dependent DM					
		Sample: 6–20 years old (Semarang, Surubaya), all children who attended the clinic between 1973–1988 aged between 1–15 years old (clinic)			

Notes: OGTT: Oral-glucose tolerance test; FPG: Fasting plasma glucose.

A very high prevalence of diabetes mellitus (19.6%) was found in the suburban population of Ternate, a small remote island in Eastern Indonesia in 2008 [12]. This finding supports results from *Riskesdas* 2007 which identified the North Moluccas province (to which the city of Ternate belongs), jointly together with West Kalimantan, to be the provinces with the highest rates of diabetes prevalence [16]. Marriage between close relatives is common in this area and can lead to diabetes inheritance; indeed multivariate analysis showed that a family history of diabetes was a risk factor for diabetes in the study [12].

Epidemiological studies in urban areas of Indonesia showed a marked increase of diabetes prevalence in the last 30 years. Diabetes prevalence in Jakarta rose from 1.7% in 1982 to 5.7% in 1993, and then more than doubled to 12.8% in 2001 [48]. A study in Ujung Pandang also showed similar results [48]. In contrast, a study in the rural area of Ende found a much lower prevalence of 1.56% (Soebardi 2011, unpublished observations).

This urban vs. rural divide concerning the burden of diabetes is supported by a study on the causes of death in an urban (Surakarta) and rural city (Pekalongan) in Indonesia. In this study, diabetes was identified as the second main cause of death in the urban municipality (representing 8.5% of all deaths) but was not one of the main causes of death in the rural municipality [17]. At the national level, diabetes was identified as the third main cause of death after stroke and hypertension, ahead of cancer and chronic obstructive pulmonary disease [11,16,49].

In terms of risk factors, studies identified age, central obesity, hypertension and smoking habits as risk factors for undiagnosed diabetes [10] and dyslipidaemia as a risk factor in newly diagnosed patients in comparison to non-diabetic patients [43]. Although no formal statistical testing was conducted, prevalence of diabetes increased with age, was higher in women, people with no primary education, housewives, jobseekers, followed by employees and entrepreneurs according to *Riskesdas* 2007 [16]. This finding seems to indicate that diabetes affects the less affluent and the affluent alike. However, in the same study, diabetes prevalence was found to rise with increasing levels of household expenditure per capita [16].

There is very little evidence on the prevalence of gestational diabetes mellitus in the country. One study using WHO criteria estimated a prevalence of 9.8% in 1998 (unpublished study, findings reported in [50], gestational week was not specified) and a previous study using Sullivan and Mahan criteria reported a prevalence of 1.9-3.5% (unpublished study, findings reported in [50,51], no information on reference year or gestational week).

### Diabetes costs

At the time of writing there was no published evidence on the national direct costs of diabetes. However, there are a few local studies (mostly from individual hospitals) on diabetes costs and also unpublished national data from *Askes*. In addition to that, the IDMPS study offers some insights into diabetes-related resources use. There is no published evidence on the indirect costs of diabetes.

#### ***Local studies***

A study on the costs of diabetes of 100 diabetic patients at Kodya hospital Yogyakarta (secondary health care facility) was conducted in 2004. Monthly mean direct costs of type 2 diabetes was estimated at USD$ 19.97 [19]. Most of the direct medical costs identified were spent on drugs (96.4%).

The use of triple drug combinations was found in 36% of cases and among those, the combination of glikuidone, metformin and acarbose was the most expensive regimen (USD$ 39.44)[19].

A similar study was conducted at the Dr. Sardjito hospital in Yogyakarta (tertiary health care facility) in 2005. The average direct cost per month for diabetes treatment was IDR 208,500 (USD$ 21) of which 59.5% was spent on drugs, followed by 31% which was spent on diabetes-related complications [20].

The combination of biguanid, alpha glucosidase inhibitor and insulin was the most expensive drug combination at IDR 571,000 (USD$ 57) per month [20].

These two studies suggest that in tertiary care facilities costs of diabetes were high for most of the Indonesian population (average GDP per capita is USD$ 245.5 per month in 2010 [52]), particularly in a context of incomplete insurance coverage. Of course these higher costs are likely to be caused by the more complex nature of cases (e.g. presence of co-morbidities and complications) in a tertiary level hospital.

An ongoing study is trying to estimate diabetes-related costs in Cipto Mangunkusumo National Public Hospital (RSCM), a national referral hospital located in Jakarta. Preliminary results indicate that diagnostic costs for comprehensive evaluation (consultation fee and further examinations) are about USD$ 150 (Tahapary 2011, unpublished data).

#### ***Askes data***

In 2010, *Askes* covered around 16.5 million people in Indonesia (7% of the population). In the same year, diabetes was the second most common diagnosis made (420,743 people) after hypertension. It was reported that *Askes* covered diabetes treatment costs of more than USD$ 22.4 million in 2010. The yearly treatment cost for diabetics without chronic kidney disease was estimated around USD$ 40, while in patients with complications, the cost can be as high as USD$ 800 (*Askes* 2011, unpublished data).

#### ***International diabetes management practices study***

This study reports the annual quantities of diabetes-related resource use in different countries from 2006–07. In Indonesia, the annual number of specialist visits was higher (10.2, SD 7.5) than GP visits (6.2, SD 7.8), while the annual number of diabetes educator visits was 1.4 (SD 2.8) [18].

### Diabetes complications

Published studies on the prevalence of complications in diabetic patients show that the most frequent diabetic complications were: neuropathy (78-13%), albuminuria (77.7-33%), microvascular complications (53–27.6%), decreased glomeral filtration rate (43.7-7.5% varying also depending on the method used), retinopathy (42.6-17.2%), nephropathy (26–7.3%), macrovascular complications (20-16%), and diabetic foot (24–7.3%) (Table 3 and Table 4).

**Table 3:** Complications of the vascular system

**Estimate**	**Study year**	**Sample frame and sample**	**Study design**	**Diagnostic test and diagnostic criteria**	**Reference**
**Diabetic retinopathy**					
42.6%, n = 760, N = 1785	Nov 2008 - Feb 2009	Sample frame: 18 diabetes centres, nationally representative	Cross-sectional study	Blood samples	[26]
		Sample: 1785 DM T2 patients, ≥ 15 years old			
17.2%, n = 52, N = 302	Year: NA	Sample frame: Diabetes clinic in Surabaya	Retrospective review of medical records over one year		[28]
		Sample: 302 T2DM patients, 132 males and 170 female, mean age of 55.9 ± 21.1 years			
28%	2002	Sample frame: Primary health care, Jakarta (Urban Area)	Cross sectional study		[27]
		Sample: NA, 30–60 years old			
**Diabetic neuropathy**					
63.5%, n = 1133, N = 1785	Nov 2008 - Feb 2009	Sample frame: 18 diabetes centres, nationally representative	Cross-sectional study	Blood samples	[26]
		Sample: 1785 DM T2 patients, > = 15 years old			
58.6%, n = 177, N = 302	Year: NA	Sample frame: Diabetes clinic in Surabaya	Retrospective review of medical records over one year		[28]
		Sample: 302 T2DM patients, 132 males and 170 female, mean age of 55.9 ± 21.1 years			
70% in malnutrition-related diabetes, n = 7, n = 10	Study period: NA	Sample frame: Dr. Sutomo Hospital, Surabaya	Cross-sectional		[31]
		Sample: 27 diabetic patients between 22 and 55 years old			
78% in non-insulin-dependent diabetes, n = 7,					
N = 9 13% in insulin-dependent diabetes, n = 1, N = 8					
**Vascular complications**					
Macrovascular 16%, n = 285; microvascular 27.6%, n = 493; N = 1785	Nov 2008 - Feb 2009	Sample frame: 18 diabetes centres, nationally representative	Cross-sectional study	Blood samples	[26]
		Sample: 1785 DM T2 patients, > = 15 years old			
Macrovascular 20% microvascular 53%	2006 - 2007	Sample frame: Patients with type 2 diabetes	Multi-centre, cross-sectional, observational study.	Prevalence-based approach to estimate resource use occurred during a 1-year period.	[18]
		Sample: N = 674, mean age 55.2 (SD = 10.2), 55% females, mean duration for diabetes 6.1 (SD = 6.4) years.			
Diabetic foot 7.3%, n = 22, N = 302	Year: NA	Sample frame: Diabetes clinic in Surabaya	Retrospective review of medical records over one year		[28]
		Sample: 302 T2DM patients, 132 males and 170 female, mean age of 55.9 ± 21.1 years			
Peripheral arterial disease (PAD) DM type 2 patients	Year: NA	Sample frame: 14 hospitals in Indonesia DM type 2 diabetes patients, aged 50 and older with one or more of the following risk factors: on smoking or with history of smoking, hypertension, dyslipidaemia		Blood pressure measurement at upper arms and ankles to obtain the ankle-brachial index (ABI)	[23]
13,807 PAD patients per 100,000 patients					
PAD was defined as an ABI value lower than 0.9.		Sample: 464 males and 521 females			
Prevalence of diabetic foot according to Wagner’s classification	1999 - 2004	Sample frame: Koja Regional General Hospital Jakarta	Retrospective analysis of medical records.		[24]
Degree 0 (high risk-foot with no ulcer) 71.6% (n = 202)		Sample: Diabetic patients, inpatient and outpatient, with diabetic foot			
Degree 1 (superficial ulcer) 1.8% (n = 5)					
Degree 2 (deep ulcer with no bone involvement or abscess formation) 2.5% (n = 7)					
Degree 3 (deep ulcer with cellulitis or abscess formation) 3.9% (n = 11)					
Degree 4 (localised gangrene) 6.7% (n = 19)					
Degree 5 (extended gangrene involving the whole foot) 13.5% (n = 38)					
As many as 15% of diabetic patients will suffer from ulcer in their lifetime, and will 12-24% undergo amputation.	2007	Sample frame: Tertiary Care Hospital	Retrospective study		(unpublished observations^i^)
		Sample: Sequential sampling of all diabetic patients hospitalised in Cipto Mangunkusumo Hospital			

**Table 4:** Complications of the renal system

**Estimate**	**Study year**	**Sample frame and sample**	**Study design**	**Diagnostic test and diagnostic criteria**	**Reference**
**Diabetic nephropathy**					
7.3%, n = 131, N = 1785	Nov 2008 - Feb 2009	Sample frame: 18 diabetes centres, nationally representative	Cross-sectional study	Blood samples	[26]
		Sample: 1785 DM T2 patients, > = 15 years old			
19.2%, n = 58, N = 302	Year: NA	Sample frame: Diabetes clinic in Surabaya	Retrospective review of medical records over one year		[28]
		Sample: 302 T2DM patients, 132 males and 170 female, mean age of 55.9 ± 21.1 years			
Overt nephropathy 11% Incipient nephropathy 26%	2003	Sample frame: Tertiary care hospital, Outpatient Endocrinology Clinic	Cross sectional study		[30]
		Sample: 100 consecutive sampling, mean age 54 (SD 9.6) years old, 42% male 58% female			
Overt nephropathy 8% Incipient nephropathy 25%	2002	Sample frame: Primary health care, Jakarta (Urban Area)	Cross sectional study		[27]
		Sample: NA, 30–60 years old			
**Chronic kidney disease**					
Prevalence of decreased glomeral filtration rate (GFR < 60 ml/min) in newly diagnosed patients with type-2 DM:	Jan 2003-Dec 2006	Sample frame: Outpatient Endocrinology clinic, Cipto Mangunkusumo hospital	Retrospective study		[25]
		Sample: 1283 new diagnosed DM type 2 patients			
Cockroft-Gault (CG) All ages: 36.1% ≥ 60 years old: 54.1%					
Modification of diet in renal disease (MDRD) All ages: 13.2% ≥ 60 years old: 19.3%					
CG-adjusted to body surface (BSA) All ages: 43.7% ≥ 60 years old: 63%					
Chinese adapted MDRD (C-MDRD) All ages: 22.8%					
Creatinine > 2 mg/ml (abnormal level in Indonesia) All ages: 5.8% ≥ 60 years old: 7.5%					
Prevalence of decreased glomeral filtration rate (GFR < 60 ml/min) patients with type-2 DM:	2002	Sample frame: Primary health care, Jakarta (Urban Area)	Cross sectional study		[27]
		Sample: NA, 30–60 years old			
30.7% (n = 16, N = 52) Albuminuria: 33%					
Prevalence of decreased glomeral filtration rate	Year: NA	Sample frame: Four urban and semi-urban areas: Yogyakarta, Jakarta, Surabaya and Bali	Cross-sectional survey		[32]
Cockroft-Gault: GFR ≥ 60 ml/min 87.5% (n = 1310) GFR < 60 ml/min 12.5% (n = 187)					
		Sample: 9412 (64.1% females). Note: From Prodjosudjadi et al. 2009 it is not clear which sub-sample was used to estimate the glomeral filtration rate.			
MDRD GFR ≥ 60 ml/min 91.4% (n = 1370) GFR < 60 ml/min 8.6% (n = 129)					
C-MDRD GFR ≥ 60 ml/min 92.5% (n = 1386) GFR < 60 ml/min 7.5% (n = 133)					
Prevalence of:	May-October 2002	Sample frame: three medical centres (outpatient)			[29]
-albuminuria: 77.7%					
-macro-albuminuria: 44.7% (41.2-48.1, 95% CI)					
-micro-albuminuria: 33% (29.7-36.3, 95% CI)		Sample: 207 patients aged 18 years and older, with hypertension and DMT2			
Incidence of renal replacement therapy for end-stage renal disease in Indonesia was 14.5 (n = 2149) and 30.7 (n = 4656) per million population in 2002 and 2006 respectively.	2002-2006	Sample frame: 13 nephrology centres in public and private hospitals	Retrospective study		[22]
		Sample: Total number of patients on renal replacement therapy (either haemodyalisis, continuous ambulatory peritoneal dialysis or renal transplant)			
Prevalence of renal replacement therapy for end-stage renal disease in Indonesia was 10.2 (n = 1517) and 23.4 (n = 3549) in 2002 and 2006 respectively.					
Complications in children with DM type 1	Nov 2001 - April 2002	Sample frame: Seven diabetes centres	Cross-sectional clinic-based survey		[21]
Hypoglycaemia (Events per 100 patient-years, rate (95% CI)): 76.2 (3.4 to 149)		Sample: 64 DM type 1 patients (45% boys, 55%, girls), mean age 11			
Diabetic ketoacidosis: 20.3 (9.5 to 31.2)					
Microalbuminuria (n,%): 3 (4.7)					
Hypertension (%) 31.7					

Notes: Modification of diet in renal disease (MDRD), Cockroft-Gault (CG).

We found a similar number of studies at the hospital level [24,25,27,28,30,31] and at multiple hospital or diabetes centre locations [18,21-23,26,29,32]. However, only one study was considered to be nationally representative [26].

A study on end-stage renal disease (ESRD) in Indonesia showed increasing incidence (measured as the total number of ESRD patients undergoing renal replacement therapy per million people) and prevalence rates (measured as the total number of ESRD patients alive on December 31 of the current year per million people) of ESRD between 2002 and 2004 in East and Central Java, in Jakarta, and in Bali [34]. The exception is West Java where incidence and prevalence decreased between 2002 and 2004 [34]. Furthermore, Bali stands out with a very high increase in incidence and prevalence between 2000 and 2003 [34].

### Cost of complications

A study of type 2 diabetes patients who failed (the study does not provide a definition of failure) with oral antidiabetic medications measured the cost of patients with and without complications. In patients with both microvascular and macrovascular complications, the total cost of management increased up to 130% compared to those without complications [33]. Between 2007 and 2008, the 6-month direct medical cost among type 2 diabetic patients with no complications, one complication and two or more complications were USD$ 339.14, USD$ 433.44 and USD$ 478.8 respectively [33].

Data from a hospital-based study in 2005 showed that the highest cost for monthly treatment was recorded for patients with complications including hypertension and retinopathy of IDR 754,500 (USD$ 75) [20].

Haemodialysis (HD) imposes a high cost of treatment on most ESRD patients with limited or no insurance, who are mostly from low socioeconomic groups. The annual costs of HD twice a week was about USD$ 4,900-6,500 [34] (the year to which this estimate refers is not clear from the source but is likely to be 2000–2003) while the GDP per capita at constant USD$ (2000) was USD$ 816 in 2002 and USD$ 876 in 2004 [52]. These costs refer to the public sector and are subject to variation within the country.

The cost for renal-replacement therapy is paid as part of government health insurance and came to USD$ 5,776,565 in 2002 and to USD$ 7,691,046 in 2006 [22]. The health insurance (*Askes*, *Jamkesmas*) covers the renal replacement therapy (RRT) but with some limitations; notably, the coverage of haemodialysis is limited to two sessions a week. In addition, there are geographical barriers affecting availability of RRT units.

However, due to limited insurance coverage, a large proportion of patients have to pay out-of-pocket for HD [34,35]. Continuous ambulatory peritoneal dialysis (CAPD), an alternative treatment to HD, is offered in a limited number of centres but its costs (CAPD catheter insertion: USD$ 1,150, annual costs of four fluid exchanges: USD$ 4,800-6,400) are not fully covered by insurance, not even for Government officials [34]. The use of renal transplantation as an alternative to dialysis is still limited mainly because of religious issues regarding the use of cadaveric donors, limited number of doctors able to perform this intervention, and the financial barriers. The cost for pre-transplantation and transplantation varied between USD$ 12,000 – 15,650, while the annual cost for immunosuppressive drugs ranged between USD$ 6,250 – 10,000 [34,35].

### Treatment

A few studies have looked at the type of treatment used by patients attending diabetes centres and the use of renal replacement therapy. For diabetes type 2, one study among patients aged on average 59 years reported that most patients (61.9%, n = 1133) received oral antidiabetic drugs monotherapy, followed by insulin and oral antidiabetic drugs (OAD) (19.4%, n = 356), insulin monotherapy (17.3%, n = 317), no treatment (1.1%, n = 20), and herbal treatment (0.3%, n = 5) [26]. For insulin therapy the most common mean number of injections per day (mean units per day 37.8) was two (55.7%, n = 371) followed by more than two (25.1%, n = 167) and one (18.9%, n = 126) [26].

A previous study in 2003 among young diabetes type 2 patients (<18 years old) found that 42.9% of patients did not receive any medication, 28.6% received insulin and OAD combination therapy, and 14.3% received insulin monotherapy and another 14.3% received OAD monotherapy [36]. These data need to be compared with caution as the first study is nationally representative while the second study was part of a larger Western Pacific study on diabetes and the sample size for Indonesia was only seven patients.

A third study looked at the treatment regimen of diabetes type 1 patients and found that most patients received one to two injections per day (87.8%) while 10.2% received three injections per day (mean dose across all countries included in the study was 1.0 + -0.4U/kg) [21]. It is not clear what type of treatment the remaining 2% received.

The use of renal transplantation is very limited for a number of reasons which include: the costs of kidney transplantation are unaffordable for the majority of the population; cultural and religious beliefs; perception of the law; lack of information about organ donation; and lack of infrastructure and skilled health personnel [38]. Between 1997 and 2001, only 247 transplantations from living donors were performed in Indonesia in comparison to 757 in Thailand and 1246 in the Philippines both from living and cadaveric donors [37]. The use of continuous ambulatory peritoneal dialysis (CAPD) has been increasing from 23 patients in 2002, to 152 in 2004, 592 in 2006, and 774 in 2007 [35]. However, the rate of dropout, mainly due to death, infection, or catheter failure, was also reported to be high [35].

### Outcomes and control of diabetes

Evidence on the outcomes and control of diabetes was scarce. We identified two main studies in the area, DiabCare 2008 which assessed outcomes, control and complications of diabetes as well as quality of life of patients, and the IDMPS in 2006–07 [18,26] (Table 5). The quality of life data collected as part of DiabCare 2008 found that most responses from patients fell in the positive impact territory of WHO-5-wellbeing index [26]. We identified a few other multi-country studies, but they did not present all their results at the national level.

**Table 5:** Outcomes

**Estimate**	**Study year**	**Sample frame and sample size**	**Study design**	**Reference**
*DMT2*				
HbA1c 68% patients >7% 81% patients >6.5%	Nov 2008 - Feb 2009	Sample frame: 18 diabetes centres	Cross-sectional study, blood samples	DiabCare 2008 [26]
FPG 47% patients >7.2 mmol/l 69% patients >6.1 mmol/l		Sample size: 1832 participants (1785 DM T2, 17 DM T1)		
47-69% of the surveyed patients depending on the criteria applied (IDF/ADA or APDPG)				
HbA1c: 69% patients >7%	2006 - 2007	Sample frame: Patients with type 2 diabetes	Multi-centre, cross-sectional, observational study.	IDMPS [18]
		Sample size: N = 674, mean age 55.2 (SD = 10.2), 55% females, mean duration for diabetes 6.1 (SD = 6.4) years.		
			Prevalence-based approach to estimate resource use occurred during a 1-year period.	
Mean ± SD HbA1c 8.1 ± 2 (N = 1932)	Mar - Dec 1998	Sample frame: several diabetes centres with more than 100 diabetic patients a month	Cross-sectional survey	DiabCare 1998 [39]
Categories by central HbA1c values (%)				
<7: 34%		Sample size: N = 1932		
7-8: 25%				
>8: 41%				
Local mean ± SD HbA1c 7.7 ± 2.6 (N = 144)				
*DMT1*				
HbA1c: 10.5+/-2.7 (mean +/- SD)	Nov 2001 - Apr 2002	Sample frame: Seven diabetes centres	Cross-sectional clinic-based survey	[21]
		Sample size: 64 DM type 1 patients (45% boys, 55%, girls), mean age 11		
HbA1c:	Apr - Jun 1999	Sample frame: Children with type 1 diabetes at the Paediatric Endocrinology Clinic of the University of Indonesia.	Cross-sectional survey	[41]
46% patients ≤ 10				
54% patients ≥ 10				
		Sample size: N = 24 children mean age 13.6 years old 10 boys, 14 girls.		
**Annual checks for complications and glucose monitoring**				
*DMT2*				
Frequency of eye examination among known diabetic patients in the last year: 15.3%, n = 30, N = 196	Feb - Apr 2009	Sample frame: One tertiary hospital and two community clinics in Jakarta		[40]
		Sample size: N = 196, mean age 58.4 years, 61.5% females.		
Percentage screened for	2006 - 2007	Sample frame: Patients with type 2 diabetes	Multi-centre, cross-sectional, observational study	IDMPS [18]
-Micro-vascular complications: 56%		Sample size: N = 674, > 18 years with average age 55.2 (SD = 10.2), 55% females, mean duration for diabetes 6.1 (SD = 6.4) years.		
			Prevalence-based approach to estimate resource use occurred during a 1-year period.	
-Macro-vascular complications: 49%				
Home blood glucose monitoring 57.1%	2003	Sample frame: Patients with type 2 diabetes	Clinic-based survey	[36]
Four or more clinic visits in the previous year: 57.2%		Sample size: N = 7, 42.9% male, aged <18 years		
≥ 3 HbA1c test in the previous year: 16.7%				
*DMT1*				
No HbA1c test in the previous year: 37%	Nov 2001-Apr 2002	Sample frame: Seven diabetes centres	Cross-sectional clinic-based survey	[21]
More than three HbA1c test in the previous year: 21%		Sample size: 64 DM type 1 patients (45% boys, 55%, girls), mean age 11		
Blood glucose self-monitoring was performed on average 32 times a month				

Notes: Fasting plasma glucose (FPG), standard deviation (SD).

There seems to be general agreement between studies that more than 60% of diabetes type 2 patients had HbA1c levels greater than 7%. HbA1c levels were suboptimal also for diabetes type 1 patients according to two surveys conducted at clinic level; one study showed an average HbA1c level of 10.5 [21] and the other showed that more than half of the assessed patients had levels above 10 [41]. Information on the frequency of annual checks for complications and glucose monitoring is limited and only covers patients attending clinics. Available information suggests that among patients attending clinics, about half were tested for micro- and macrovascular complications (56% and 46% respectively) [18] and that more than half of the patients performed home glucose monitoring and had four or more clinic visits in the past year [36]. For patients with type 1 diabetes (though the sample size is very small), 37% did not have any HbA1c testing in the previous year, while 21% had three or more tests [21].

A study on the frequency of annual eye checks among diabetes patients in urban Jakarta found that only 15.3% of them had undergone eye examinations. Screening was found to be correlated with knowledge about diabetic retinopathy along with the number of years since diagnosis was made, and not to be correlated with education, income, health insurance status, or diagnosis of diabetic retinopathy. Respondents who did not go for annual checks mentioned lack of knowledge (60.6%) and financial barriers (13.8%) as reasons for not getting screened [40].

*Askes* in collaboration with *Perkeni* developed a training module (PROLANIS) for general practitioners and monitored their diabetes treatment. Data from PROLANIS on blood glucose control between 2010 and 2011, show that the percentage of diabetic patients reaching target levels of fasting plasma glucose (FPG) and post prandial blood glucose (PPBG) increased significantly (FBG 15% to 51%; PPBG 18% to 48%) (*Askes* 2011, unpublished observations).

### National guidelines for diabetes treatment and prevention

The Indonesian Society of Endocrinology (*Perkeni*) is responsible for developing diabetes treatment guidelines in Indonesia. Guidelines for diabetes mellitus type 2 are periodically reviewed and the latest version was last published in 2011. Screening is recommended for high risk groups such as individuals with a sedentary lifestyle, a lack of physical activity, an unhealthy diet, a family history of diabetes, obesity, hypertension, dyslipidemia, coronary artery disease, polycystic ovary syndrome, a history of gestational diabetes, and/or have given birth to a baby weighing more than 4 kg. [48]. The guidelines recommend FPG or random blood glucose only if the classic symptoms of diabetes mellitus such as polynuria, polyphagia, polydipsia and weight loss without etiology are present [48]. If classic symptoms are not present, the oral glucose tolerance test (OGTT) is recommended to be performed according to WHO recommendations [48]. For high risk individuals with a negative result, it is recommended that the test should be repeated on an annual basis, while for people aged over 45 and without other risk factors, screening is recommended every three years [48]. The guidelines also address issues related to diabetes management in relation to smoking, tuberculosis, and fasting but not in relation to HIV.

Treatment guidelines for type 1 diabetes were developed in 2000 and have been revised in the frame of a project in collaboration with the World Diabetes Foundation (2008–2011) [53]. However, at the time of writing, we could not find any publicly available version on the internet.

Additional guidelines for insulin therapy [54] and dyslipidaemia were recently published [55]. It is difficult to assess implementation of these guidelines but the Indonesian Society of Endocrinology has adopted an intense dissemination strategy by promoting the use of the guidelines through symposiums, workshops and training schemes.

### Smoking, TB, HIV and fasting

Smoking is a major problem among men in Indonesia (prevalence 61% and 5% among men and women respectively, aged 15 and older in 2009 [3]). Unfortunately, there has been very little evidence on the impact of tobacco consumption on diabetes and its complications in Indonesia. One study of 778 male diabetic patients in a clinic in Yogyakarta between 2006–07 found that 65% patients smoked before they were diagnosed [56]. The study showed that most patients were unaware that smoking could lead to serious complications; 34% thought that smoking would not aggravate diabetes; 25% did not know; and 41% thought smoking aggravated diabetes [56]. Yet interestingly, despite minimal efforts by health doctors and nurses to encourage smoking cessation, 74.4% of the patients stopped smoking since diagnosis [56].

Uncontrolled diabetes can lead to various complications including increased susceptibility to infections [57] such as TB and HIV. In turn, these infections can worsen glycaemic control [58] and therefore impact negatively on diabetes management. Additionally, there are drug-to-drug interactions which may come into play.

Despite the high prevalence of TB (281 cases per 100 000 population in 2011 [3]), there was only one study on the association between diabetes and tuberculosis in Indonesia [59]. This case–control study found prevalence of diabetes mellitus among newly diagnosed TB patients (median age 30 years, median body mass index 17.7 cases, 21.5 controls) was 13.2% and 3.2% in control subjects (OR 4.7, 95% CI 2.7-8.1) [59]. There was no study looking at the impact of these co-morbidities on diabetes management (including drug-to-drug interactions), complications, or outcomes.

No study on diabetes mellitus and HIV/AIDS was found. The majority of the Indonesian population is Muslim. A small study (n = 24) evaluating the effects of fasting on diabetes showed that Ramadan fasting can improve metabolic control by reducing serum fructosamine and beta hydroxybutirate in patients with well controlled type 2 diabetes mellitus without causing a formation of beta hyroxybutirate (which is responsible for ketoacidosis) [60].

### Programmes

The Indonesian Endocrinology Society (*Perkeni*), the Indonesian Diabetes Association (PERSADIA), and the Ministry of Health in collaboration with the World Diabetes Fund (WDF) and other partners have implemented a series of programmes to address existing challenges in diabetes management.

#### ***Ongoing***

One ongoing project aims to address the human resource capacity gap by training master level staff, nurses, educators, patients and their relatives in diabetes management between 2011 and 2014. Moreover, community members will be reached by awareness raising and educational activities [61].

Another current project aims to reduce the prevalence of NCDs and their risk factors by using a community-based approach in the provinces of West Sumatra, Bengkulu and Banten [62]. This project builds on a successful pilot project in Depot in 2001 [63] and if successful, will be integrated into the national programme for NCDs and extended to the remaining 30 provinces [62]. This project, implemented between 2010 and 2013, aims to provide early detection, counselling and education for people with or at risk of developing NCDs [62].

To date a number of results have been achieved including the development of a local policy on prevention and control of diabetes and related NCDs; lay people participation in community needs assessment; training of nurses and health workers in integrated prevention and control of NCDs; the training of teams of medical professionals in clinical NCD control; the training of cadres of health workers in risk factors; and the selection and training of counsellors as dietary educators [62]. In addition, 18 integrated community health posts (Posbindu PTM) and groups for people living with or at risk of NCDs have been established and 1,800-3,600 people have been screened, monitored and counselled on risk factors at the community health posts [62].

Another ongoing project (2009–2012) at the time of writing is trying to improve the ability of the health care system to deliver diabetes retinopathy care. The current project outcomes include the renovation of a screening facility and the purchase of diagnostic equipment; training of healthcare staff including specialists as well as educators; awareness raising activities; screening and treatment of diabetic retinopathy [64]. As part of this initiative, Cipto Mangunkusomo Hospital strengthened collaborations between the endocrinology clinic and the ophthalmology department for the treatment of diabetes retinopathy [65]. All newly diagnosed diabetes patients are referred for the DR test and existing patients are checked once a year. Screening and retinal photo grading are provided for free and vouchers are distributed in the community to encourage people to visit the hospital to get screened [65]. The cost of laser treatment varies depending on the insurance scheme but it is free for non-insured people. Furthermore, a registration card was introduced to facilitate keeping track of patients [65].

#### ***Completed***

To address the lack of trained staff in diabetes foot care and the paucity of diabetes foot clinics (only four at the beginning of the project), a training programme was conducted between 2008 and 2011 [66]. As a result of this, three internists and two nurses received intensive foot care training, who then in turn trained 40 foot care teams (40 internists and 68 nurses) from all provinces in Indonesia in basic foot care training. Additionally, 14 new diabetic foot clinics have been established and two clinics improved; 8,000 patients have been screened for diabetic foot; and training modules, guidelines and education materials have been developed [66].

In an attempt to address the lack of awareness about type 1 diabetes in children, a programme to improve management of this condition was implemented [53]. Between 2008–2011, 381 paediatricians from seven cities have been trained in the management of type 1 diabetes; 61 nurses have been trained as diabetes educators; 150 families with children having type 1 diabetes have been trained in diabetes management; 731 children with diabetes have been registered and now receive care; treatment guidelines for type 1 diabetes have been revised; and nearly 11 million people have been reached through media awareness activities [53].

Between 2006 and 2008, 1,237 health care professionals have been trained in diabetes management in eight cities/provinces (Jakarta, Yogyakarta, Surabaya, Denpasar, Medan, Makassar, Bandung and Padang) in order to improve diabetes care delivery [67]. This led to an increase in the percentage of hospitals providing diabetes education from 52.8% to 67.7% and from 46.3% to 67.8% in primary health centres [67].

Lack of awareness about the disease is particularly problematic in rural areas. This can lead to delayed diagnosis and early onset of complications. To address this, but also to increase the ability of healthcare workers to meet the needs of patients, a public awareness raising and training for healthcare staff was implemented between 2005–2008 in two rural areas (Kediri City and Kediri Regency). During this time period, social educators and health workers have been trained and diabetes awareness information posts have been established in 26 districts [68].

#### ***PROLANIS***

Prolanis is a chronic diseases management programme that is part of *Askes*. The programme started in 2010 and focuses on the self-management of diabetes. It shifted part of the consultation services and monthly checks from the hospital to the health centre to benefit patients in terms of significantly lower waiting times and more time for counseling and patient education [69]. This is a positive change for *Askes* insurees but raises questions of unequal access to information and education for those not insured by *Askes*.

### Policy and strategy to address diabetes

Indonesia has released its first diabetes programme at the National Congress of Perkeni in July 2012. The programme will include a variety of stakeholders involved in diabetes management and will focus on prevention and increasing the capacity for diagnosis and management of diabetes. In addition to that, an extensive training programme for doctors in the field of diabetes is currently running.

## Discussion

A key focus area that needs to be addressed is the current lack of formulated health policies, strategies and action plans to tackle the emerging diabetes epidemic. Isolated interventions do take place, but in the absence of an overall framework guiding the process and ensuring sustainability, planning, and coherence, its overall impact may suffer. On the positive side, a national diabetes plan was launched in late 2012.

### Health systems challenges in diabetes management

This section reflects on health system challenges from some of the authors’ own experiences of working in the country.

The provision of quality care for diabetes patients starting from early diagnosis to treatment and prevention of complications in Indonesia is hampered by a fragile health system. The health workforce has major deficiencies in terms of numbers and quality of training, greatly affecting the quality and efficiency of health service in Indonesia. In 2007, the number of physicians per 10,000 population was 2.9, well below the regional average (5.6) and the world average (14.2), while the number of nurses and midwives per 10,000 population was 20.4, higher than the regional average (10.9) but below the world average (28.1) [3]. In terms of specialists, in 2010, there were only about 70 endocrinologists in the country [70]. Moreover, most general practitioners and midwives work in urban areas and only a limited number of them practice in remote areas.

In terms of public infrastructure at the primary care level, Indonesia is generally regarded as having relatively adequate levels of provision with one public health centre for every 30,000 people on average [71]. However, this figure conceals large variations in geographic accessibility, with people in remote interior or small island locations having particularly poor access.

Due to the lack of expertise and diagnostic equipment at the primary care level, diabetes care is concentrated in diabetes clinics at secondary and tertiary care levels leading to higher costs for the healthcare system and the individual which can lead to increased barriers to accessing care. In this context, it is hoped that efforts towards reaching universal health coverage by 2014 will help strengthen primary care capacity particularly in the area of diabetes. The private sector is increasingly important in the provision of health care in Indonesia, especially in large cities, where there are wide variations in quality of care. Furthermore, owing to lack of regulation on pricing and quality of services, users are more susceptible to excessive treatment and overcharging [72].

Lack of resources in the public sector can partly be explained by low public spending on health. In 2010, total health expenditure on health represented only 2.6% of GDP corresponding to a total expenditure on health per capita of US$ 77 at the 2010 exchange rate (US$ 112 at purchasing power parity) [3]. Nearly half of it, 49.1%, was publicly funded [73]. Further, health insurance remains limited in coverage, breadth and depth (number of people insured and services covered).

Medicines to treat diabetes are not reaching everyone due to limited affordability and availability, as well as other factors. Medicines are generally available in main cities or in the private sector but there are serious availability problems in public primary health centres and in rural areas. At primary care level, drug availability is very limited; at hospital level, availability varies widely. This is caused mainly by the geographical barriers or supply chain problems due to underfunding. In addition, not all medicines to treat diabetes are covered by health insurance, especially the newest and more expensive classes of medicines. At the same time, the use of traditional medicines is widespread; this is particularly problematic due to the weak quality control mechanisms in place [72].

These issues are further exacerbated by a weak health information system hampered by poor coordination and integration between different data sources, duplication of data collection efforts, ill-defined division of reporting responsibilities and very little reporting from the private sector which represents half of the total service delivery [4,72]. These challenges were severely aggravated by the decentralisation process which led to the partial collapse of the health information system [4,72]. As a result, no nationally representative data on health indicators has become available since 2001 [4,72].

Without sound data, it is difficult to inform health policy and programmes. Although diabetes mellitus type 2 is gaining importance on the national health agenda, a coordinated framework of action for Government, donors, and the private sector is yet to be developed and implemented.

### Further considerations on the data

Nearly all the studies reviewed were conducted at health facility level. In a country like Indonesia, where incomplete insurance coverage affects access to healthcare for those in lower socioeconomic groups who are not insured and have limited ability to pay out-of-pocket, it is doubtful whether these data represent the actual incidence, prevalence, screening frequency and outcome situation in the country.

In this respect, Riskesdas differs from the studies just described, as it looks at the percentage of patients in the community who were receiving treatment at the time of the survey.

In general, only cross-sectional information is available. No longitudinal study following a cohort of patients and investigating risk factors for poor outcomes was found.

Despite the development of a national renal database, no recent study was found in the peer-reviewed literature, raising questions about the use of the data collected to inform policies and programmes. The latest available estimates from the Indonesian Society of Nephrology showed a general increase in ESRD incidence and prevalence rates between 2002 and 2006 in East and Central Java, Jakarta and Bali. The increase was probably due to improved availability of health facilities, experts and coverage through *Askes* and *Jamkesmas*.

Moreover, there was no information available on care seeking pathways or the role of the traditional sector, which is known to be important especially in rural and remote areas. There was also no information on compliance and reasons for non-compliance which are all essential pieces of evidence to inform an effective national strategy to address diabetes.

There is a lack of information on the impact of programmes beyond intermediate outcomes such as the number of people trained; percentage of health centres providing education; or development of training material and guidelines (e.g. has detection rate increased as a result of training, have outcomes improved, are there fewer complications arising due to better screening?)

For example, based on the available information online, a number of questions and concerns have arisen regarding the diabetic retinopathy initiative at Cipto Mangunkusomo Hospital. In which communities are the screening vouchers being distributed and who uses them? Are the vouchers reaching those who are most in need? Who pays for these free screening services? Who pays for the free laser treatment for uninsured patients and how should this service be sustainably financed in the long-term if more and more uninsured people start to ask for it? Will the achievement of universal health coverage be delayed? Further, are there plans to expand this initiative to other hospitals? At the moment only people living within a reasonable travelling distance from the referral hospital are able to benefit from these free screening services.

## Conclusions

Despite methodological limitations affecting the studies reviewed, evidence suggests that the prevalence of diabetes in Indonesia has increased over time. Our findings also highlighted wide disparities in the prevalence of diabetes across the country and the presence of a very large number of undiagnosed patients.

The paucity of available data (particularly for type 1 diabetes) and its unrepresentativeness of the whole country, calls for more evidence to be collected on the direct and indirect cost of diabetes. Such evidence is needed to uncover existing disparities within the country and to estimate the resources that will be needed to provide comprehensive diabetes care as part of the Government’s plan to provide universal health coverage.

Access to preventive and curative services is further challenged by disparities in health service provision, human resource distribution, and treatment availability. These disparities appear to be strengthened by the archipelago formation of the country which is conducive to unequal distribution of services and tends to favour urban areas over rural and remote areas.

In addressing these issues, the Ministry’s plan to reach universal health coverage represents an opportunity to strengthen access to healthcare and the number and the quality of services offered at primary care level. However, it must ensure that increased access also focuses on marginalised communities and that it will be backed by the necessary funds to ensure its scaling up and long-term sustainability.

In the light of these challenges, the authors suggest the following priority actions:

• Building on the initial positive results achieved by *Askens/Perkeni*, to improve training of primary health care doctors and nurses to include management of non-communicable diseases with particular emphasis on diabetes and its related comorbidities and complications

• To reform referral patterns from the current focus on inpatient care to a strong primary health care system

• To increase coverage of essential drugs as part of universal health insurance

• To improve availability of equipment in public primary health care facilities

• To improve health service delivery to remote areas by sharing lessons with other countries experiencing the similar challenges

• Provide education on self-management of diabetes to patients and emphasis on the importance of undergoing regular checks for complications

• To increase awareness in the population about NCDs by launching mass information campaign through main national media and education of pupils in schools

• To focus on prevention and develop strong anti-tobacco legislations and food policy to protect vulnerable groups such as children

• To strengthen data collection systems for type 1 and 2 diabetes by collecting routine data at local level; centralising them at national level for analysis; using these data, complemented by regular surveys such as Riskesdas, to inform planning and decision-making

## Endnotes

^i^Yunir, E. 2008. Diabetic Foot in Indonesia. Presentation at the Kyoto Foot Meeting. Training of Diabetic Foot Care for Young Doctors. 5–7 March 2008.

## Abbreviations

CAPD: Continuous ambulatory peritoneal dialysis; ESRD: End-stage renal disease; NCDs: Non communicable diseases; CDs: Communicable diseases; FPG: Fasting plasma glucose; Perkeni: Perkumpulan endocrinologi Indonesia; IDMP: International diabetes management practices study; IDR: Indonesian Rupee; ISE: The Indonesian society of endocrinology; Askes: Asuransi Kesehatan; Jamkesmas: Jaminan Kesehatan Masyarakat; OGTT: Oral-glucose tolerance test; TB: Tuberculosis.

## Competing interests

The funding to conduct this study was provided by Novo Nordisk Switzerland. The sponsor had no involvement in the study design, data collection and analysis, and writing. AF received travel reimbursement and speaker fees from Novo Nordisk for delivering two presentations on diabetes in EU5 at national diabetes conferences in Portugal and Spain.

## Authors’ contribution

PS retrieved data from the Ministry of Health Republic of Indonesia and *Askes*. All authors contributed to the literature review and in the appraisal of the retrieved information. PS and DT wrote the first draft, AF redrafted subsequent versions of the article up to its final version. All authors approved the final manuscript.

## Authors’ information

Pradana Soewondo currently serves as President of The Indonesian Society of Endocrinology. In this role, he provides leadership of scientific and medical activities, including research programs, professional publications, medical information, and professional education. He received his doctoral degree from Faculty of Medicine University of Indonesia. In addition to his role, Professor Pradana holds an academic appointment as a Manager of Education and Student Affairs at the Faculty of Medicine University of Indonesia. He is currently a staff member at the Division of Metabolism and Endocrinology, Department of Internal Medicine, Faculty of Medicine University of Indonesia.

Dicky Levenus Tahapary is also a staff member at the Division of Metabolism and Endocrinology, Department of Internal Medicine, Faculty of Medicine University of Indonesia and also a member of The Indonesian Society of Endocrinology.

Alessandra Ferrario is a Research Officer in health and pharmaceutical policy within the Medical Technology Research Group, LSE Health and a PhD candidate in the Department of Social Policy at the London School of Economics and Political Science.
